# Correlation of internal carotid artery diameter and carotid flow with asymmetry of the circle of Willis

**DOI:** 10.1186/s12883-020-01831-z

**Published:** 2020-06-20

**Authors:** Te-Chang Wu, Tai-Yuan Chen, Ching-Chung Ko, Jeon-Hor Chen, Ching-Po Lin

**Affiliations:** 1 Department of Biomedical Imaging and Radiological Sciences, National Yang-Ming University, Taipei, Taiwan; 2 Department of Medical Sciences Industry, Chang Jung Christian University, Tainan, Taiwan; 3 Department of Medical Imaging, Chi-Mei Medical Center, Tainan City, Taiwan; 4 Graduate Institute of Medical Sciences, Chang Jung Christian University, Tainan, Taiwan; 5 Center of General Education, Chia Nan University of Pharmacy and Science, Tainan, Taiwan; 6 Department of Radiology, E-DA Hospital, E-DA Cancer Hospital, I-Shou University, Kaohsiung, Taiwan; 7 Center for Functional Onco-Imaging of Radiological Sciences, School of Medicine, University of California, Irvine, California USA; 8 Institute of Neuroscience, School of Life Science, National Yang-Ming University, Taipei, Taiwan

**Keywords:** Cerebral blood flow, Carotid artery, MR angiography, Cerebral hemodynamics, Neuroanatomy, Ultrasound

## Abstract

**Background:**

The purpose of this study was to clarify the effect of asymmetric COW variants on carotid flow changes, and proposed an easy estimate of the representative carotid flow volume for accurate numerical simulation.

**Methods:**

A total of 210 healthy adults receiving magnetic resonance angiography and carotid duplex sonography were included. Three anterior cerebral artery asymmetry (AA) groups were defined based on the diameter ratio difference (DRD) of bilateral A1 segments: AA1 group, one-side A1 aplasia; AA2, A1 DRD ≥ 50%; AA3, A1 DRD between 10 and 50%. Similarly, 3 posterior communicating artery (PcomA) asymmetry (PA) groups were defined: PA1 group, one fetal-origin posterior cerebral artery and absent contralateral PcomA; PA2, PcomA DRD ≥ 50%; PA3, PcomA DRD between 10 and 50%.

**Results:**

With A1 asymmetry, the ICA diameter of the dominant A1 is significantly greater than the contralateral side. Significant differences of bilateral ICA flow were present in the AA1 and AA2 groups (mean flow difference 42.9 and 30.7%, respectively). Significant bilateral ICA diameter and flow differences were only found in the PA1 group. Linear regression analysis of ICA diameter and flow found a moderately positive correlation between ICA diameter and flow in all AA groups, with a 1 mm increment in vessel diameter corresponding to a 62.6 ml increment of flow volume. The product of bilateral ICA diameter and flow volume difference (ICA-PDF) could be a potential discriminator with a cutoff of 4.31 to predict A1 asymmetry ≥50% with a sensitivity of 0.81 and specificity of 0.76.

**Conclusions:**

The study verifies that A1 asymmetry causes unequal bilateral carotid inflow, and consequently different bilateral ICA diameters. Adjustment of the inflow boundary conditions according to the COW variants would be necessary to improve the accuracy of numerical simulation.

## Background

In patients with cerebral aneurysms, numerical simulation could provide important hemodynamic information about the aneurysm formation, enlargement, and rupture [1, 2]. Accurate computation fluid dynamic calculations are based on 2 basic conditions: 1) detailed 3-dimensional (3D) angio-architecture and 2) accurate/reasonable physiologic setting of the target vascular tree [2–4]. However, essential patient-specific physiologic data, such as flow volume and flow rate of the proximal parent artery, are usually lacking for numerical analysis. Consequently, average blood flow data from the general population, rather than patient-specific values, are used [5–8].

It is reported that approximately half of the population has Circle of Willis (COW) variants [9–12]. On the other hand, cerebral aneurysms are not uncommon in patients with asymmetric COW variants, such as anterior communicating artery (AcomA) aneurysms in A1 aplasia, and posterior communicating artery (PcomA) aneurysms in fetal posterior cerebral artery (F-PCA) [13, 14]. Moreover, carotid flow contralateral to A1 aplasia, and ipsilateral to F-PCA, has been shown to be greater than those with a normal COW [15–18]. Total brain blood flow and distribution in different COW-types also had been proposed [16]. It had been shown that inflow boundary condition [3, 19], especially parent artery flow volume and the conjoint inflow ratio, affect the hemodynamic parameters of cerebral aneurysms. Therefore, adjustment of inflow boundary conditions for cerebral aneurysm analysis according to different COW types is important for obtaining reliable simulation results.

Thus, the purpose of this study was to investigate the association of bilateral ICA flow in healthy adults with asymmetric COW variants, and develop a method for predicting COW asymmetry and the representative carotid flow volume.

## Methods

### Patients

This retrospective study was approved by the Institutional Review Board of our hospital. The requirement to obtain informed consent was waived due to its retrospective nature.

We retrospectively reviewed the PACS of our institution for subjects who received simultaneous head and neck MRA and carotid duplex sonography from January 2017 to June 2018 as part of a routine health examination. A total of 223 healthy subjects without any history or symptoms of cerebrovascular disease were identified.

Based on the imaging studies, 3 subjects were excluded due to segmental narrowing (> 50%) of the common/internal carotid arteries, 4 due to proximal anterior cerebral artery (ACA)/ middle cerebral artery stenosis, 1 due to a persistent trigeminal artery, 2 due to vascular anomalies such as cerebral aneurysm, 2 due to arteriovenous malformation/fistula, and 1 due to a moyamoya syndrome. These 13 excluded subjects were used for the interobserver reliability test. Thus, 210 subjects (133 males, 77 females; mean age 54 ± 9 years; range, 31 to 76 years) were included in the analysis.

### Imaging studies

Three-dimensional time-of-flight magnetic resonance angiography (TOF-MRA) of the head was obtained with the parameters: TR/TE, 21/2 ms; flip angle, 20°; FOV, 200 mm; matrix, 320 × 192; NEX, 1. The major extracranial arteries in the neck were visualized by contrast-enhanced MRA on a 3 T MR scanner (Discovery MR 750, GE Medical Systems, Milwaukee, WI, USA) using a single dose (0.1 mmol/ kg; 5–7 ml) of gadobutrol (Gd-BT-DO3A, Gadovist™, Bayer Healthcare, Leverkusen, Germany) with an injection rate of 1.5 ml/s and MR parameters of TR/TE, 4/1 ms; flip angle, 25°; FOV, 300 mm; matrix, 320 × 224; and NEX, 1. Carotid Doppler sonography was performed by the same technician with more than 10 years of experience using a Philips HD15 ultrasound system to evaluate bilateral ICA flow volume. For ICA flow volume measurement, a straight ICA segment at least 2 cm above the carotid bulb was selected with the doppler angle of incidence adjusted at or below 60 degrees. At the same site, the sample volume box was put to cover the entire vessel diameter (d). The angle-corrected time-average flow velocity (TAV) was determined over 3 to 5 complete cardiac cycles. The ICA flow volume was calculated as the product of TAV and the cross-sectional area (A) of the vessel according to the formula FV = TAV x A = TAV x [(d/2)^2^xπ].

### MR imaging interpretations

**Fig. 1:**
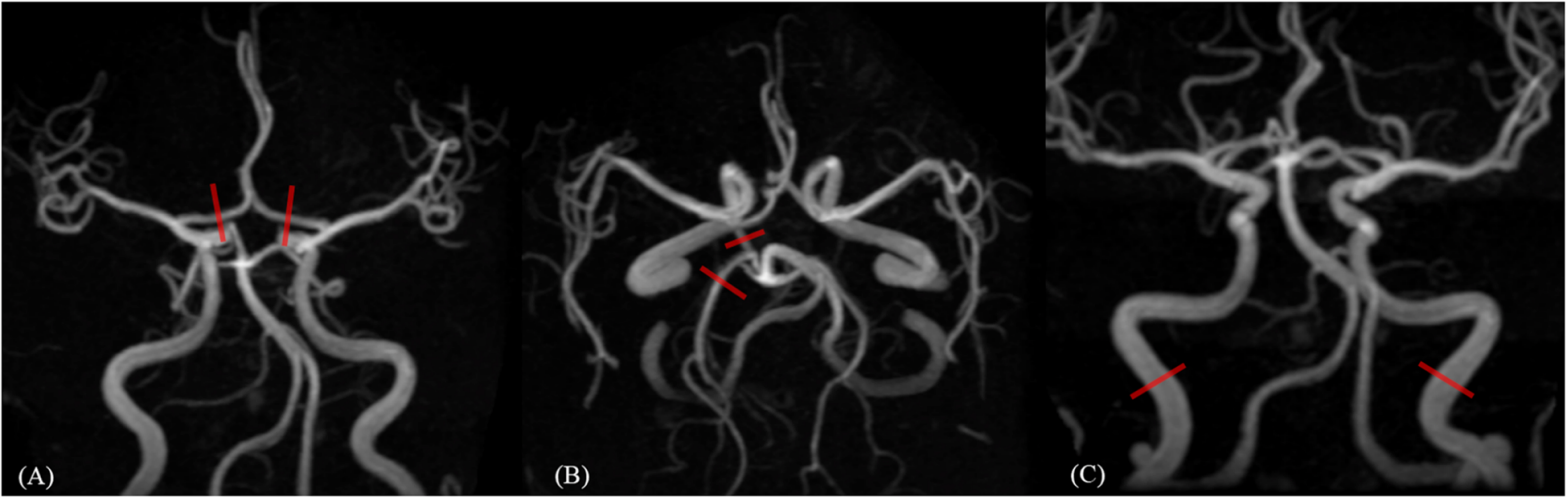
Examples of vascular diameter measurement. **a**) Typical location of bilateral A1 diameter measurement at in the middle of the A1 segment. **b**) Typical location of PcomA and P2 diameter measurement at in the middle of the PcomA and proximal P2 segment, respectively. Note there was no PcomA on the contralateral side. **c**) Typical location of bilateral distal cervical ICA diameter measurement at 1 cm below the petrous ICA segment

At first, vessel diameter and COW calcification of the 13 excluded subjects were recorded by two experienced neuroradiologists (TCW and TYC with 12 and 17 years of experience, respectively) for the interobserver reliability tests. Due to substantial to almost perfect reproducibility (ICC values of 0.82–0.97), the vessel diameter measurements of all 210 included subjects were completed by only one reader (TCW). For COW classification, the diameter ratios between the bilateral A1 segments and between the ipsilateral PcomA and P2 segments were recorded (Fig. 1a, b). The diameters of the bilateral distal cervical ICAs were measured at 1 cm below the petrous segment of the ICA (Fig. 1c). In each case, the diameter of the dominant A1 segment was set as 100%, and the diameter of the non-dominant A1 segment was set as the percentage compared to the dominant A1 segment. Bilateral PcomA diameters were transformed into the percentage of the ipsilateral P2 segment diameters. F-PCA was defined as a PcomA diameter equal to the ipsilateral P2 segment diameter with an absence of the ipsilateral P1 segment.

**Fig. 2:**
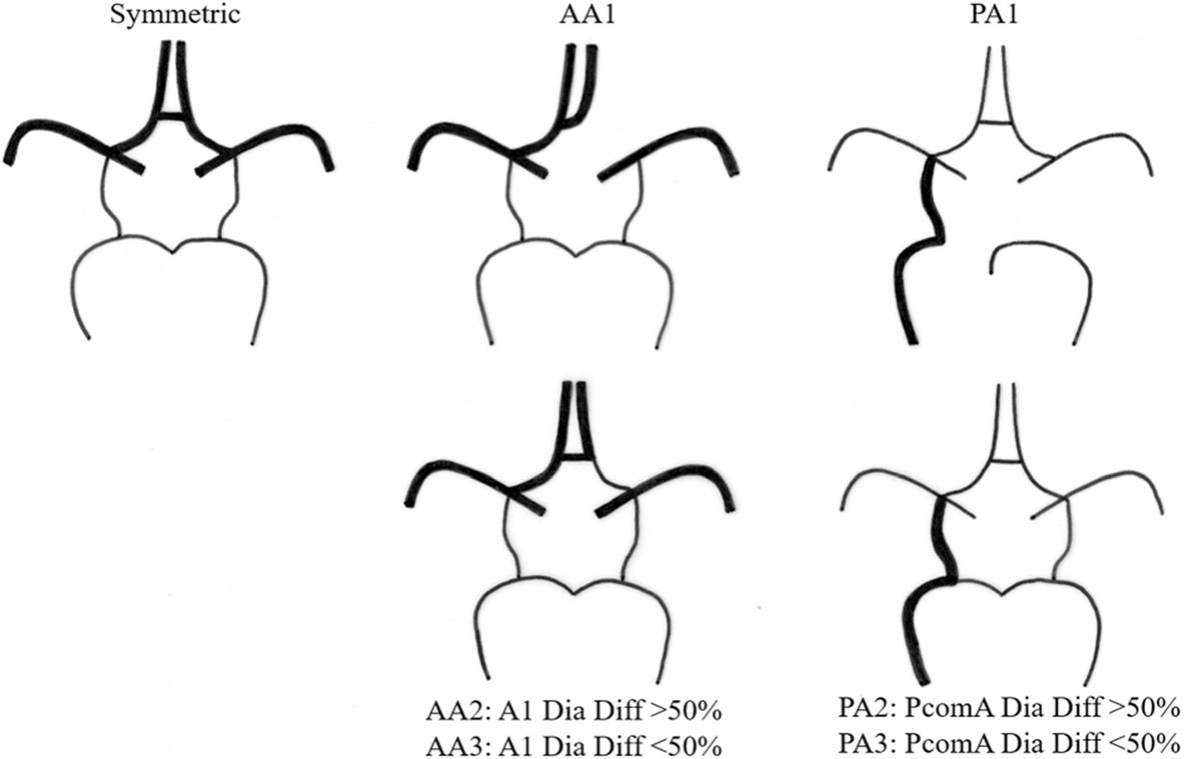
Classification of Circle of Willis variants (modified from Krabbe-Hartkamp, Radiology 1998) [9]

The classification of COW variants is summarized in Fig. 2. Three ACA asymmetry (AA) groups were defined based on the diameter ratio difference of bilateral A1 segments: AA1 group, 1 side A1 aplasia; AA2, bilateral A1 diameter ratio difference ≥ 50%; AA3, bilateral A1 ratio difference between 10 and 50%. Similarly, 3 PcomA asymmetry (PA) groups were defined: PA1 group, 1 F-PCA, and absent PcomA on the contralateral side; PA2, bilateral PcomA diameter ratio difference ≥ 50%; PA3, bilateral PcomA diameter ratio difference between 10 and 50%. According to the vessel diameter, bilateral A1 segments and PcomAs in each patient were denoted as a dominant or non-dominant side. Symmetry was defined as both bilateral A1 segment and PcomA diameter ratio differences < 10%, with the exclusion of cases with bilateral F-PCAs.

Several parameters were used to determine the ability of ICA diameter and flow volume for prediction of COW variants: (1) Bilateral ICA diameter difference; (2) Bilateral ICA flow volume difference; (3) Bilateral ICA flow volume difference percentage (2× bilateral ICA flow difference/ bilateral ICA sum); (4) Product of bilateral ICA diameter and flow volume difference (ICA-PDF), expressed as ICA diameter difference (right ICA diameter – left ICA diameter) × ICA flow volume difference percentage [2 × (right ICA flow - left ICA flow)/(right ICA flow + left ICA flow)].

### Statistical analysis

Vessel diameter and flow volume measurements were expressed as mean and standard deviation. Vessel diameter and flow volume were compared in the following ways with different statistical tests: 1) One-way ANOVA and Bonferroni method for multi-intergroup analysis; 2) Student t-test for comparisons between each AA/PA subgroup and symmetric group; 3) Paired t-test for intragroup analysis of each subgroup to compare dominant vs. non-dominant side and right vs. left side. Linear regression analysis was used to examine the relations between ICA diameter and flow volume in each COW type. To examine the ability of ICA diameter and flow parameters for prediction of each COW variant, receiver operating characteristic (ROC) curve analysis was performed. Inter-observer reliability for COW calcification of 13 excluded cases was determined by using the Cohen κ coefficient. For continuous data, the intraclass correlation coefficient (ICC) was calculated with the two-way random model and absolute agreement on average measures. The Cohen κ and ICC were interpreted according to methods described by Landis et al. [20]. Cohen K coefficient values of 0.85 ~ 0.96 were obtained for categorical COW classification and ICC values of 0.82–0.97 were obtained for the continuous data, both indicating almost perfect reproducibility. All data analyses were performed using the statistical software package SPSS for Windows version 24.0 (IBM, Chicago, IL, USA). Values of *P*-value < 0.05 were considered to indicate statistical significance.

## Results

### Vascular anatomy

The demographic data of the subjects are summarized in Table 1. All 210 subjects were asymptomatic Han-Chinese adults, and only 81 (38.6%) had complete symmetric of the COW. Seven subjects with bilateral F-PCAs and symmetric A1s were not included in the symmetric group due to a lack of bilateral P1 segments. Sixty-seven subjects had asymmetric A1 segments (31.9% of all cases), including 19 (9%; AA1 group) with unilateral A1 aplasia, 33 (15.7%; AA2 group) with ≥50% A1 asymmetry, and 15 (7.1%; AA3 group) with < 50% A1 asymmetry.

**Table 1:** Subjects demographic data by Circle of Willis variants

	**All**	**Symmetric**^**a**^	**A1 Asymmetry (AA group)**^**b**^				**PcomA Asymmetry (PA group)**^**c**^			
			**AA1**	**AA2**	**AA3**	***P*-value**	**PA1**^**d**^	**PA2**	**PA3**	***P*-value**
Number of cases	210	81 (38.6%)	19 (9%)	33 (15.7%)	15 (7.1%)		15 (7.1%)	45 (21.4%)	18 (8.6%)	
Age (years)	53.9 ± 9.4	50.4 ± 9.3	52.6 ± 11.5	54.6 ± 10.1	49.9 ± 8.3	0.344	55.4 ± 7.9	53.3 ± 9.5	51.8 ± 7.0	0.507
Right-side			6 (31.6%)	8 (24.2%)	7 (46.7%)	0.311	10 (66.7%)	29 (64.4%)	14 (77.8%)	0.588
Sex										
Female	77 (36.7%)	32 (39.5%)	7 (36.8%)	8 (24.2%)	4 (26.7%)	0.601	7 (46.7%)	15 (33.3%)	10 (55.6%)	0.262
Male	133 (63.3%)	49 (60.5%)	12 (63.2%)	25 (75.8%)	11 (73.3%)		8 (53.3%)	30 (66.7%)	8 (44.4%)	
Height (cm)	165.2 ± 8.1	165.2 ± 8.7	166.7 ± 8.2	165.5 ± 6.6	166.1 ± 6.1	0.841	163.3 ± 7.8	165.6 ± 8.7	163.4 ± 7.6	0.494
Weight (Kg)	68.0 ± 13.2	69.6 ± 14.6	69.4 ± 13.7	65.8 ± 9.9	69.7 ± 13.7	0.428	64.9 ± 11.4	67.9 ± 13.5	64.5 ± 11.0	0.543
Heart rate (/min)	67.3 ± 10.0	66.8 ± 11.1	65.8 ± 10.4	68.3 ± 8.0	65.5 ±12.6	0.551	67.8 ± 8.2	68.4 ± 7.9	69.7 ± 12/0	0.820

^a^Seven subjects with bilateral F-PCAs were excluded

^b^Twenty-three patients in the AA group had PcomA asymmetry, including 2 PA1, 14 PA2, and 7 PA3

^c^Twenty-three patients in the PA group had A1 asymmetry, including 5 AA1, 13 AA2, and 5 AA3

^d^Thirty-seven subjects with 45 F-PCA, with 20 right-side F-PCA, 9 left-side F-PCA, and 8 bilateral F-PCAs

Seventy-eight subjects (37.1% of all cases) were included in the PA group, including 15 in the PA1 group (7.1%) with a unilateral F-PCA and absent contralateral PcomAA, 45 in the PA2 group (21.4%) with ≥50% PcomA asymmetry, and 18 in the PA3 group (8.6%) with < 50% PcomA asymmetry. Twenty-three subjects had both A1 and PcomA asymmetry. In these subjects, the dominant A1 was most likely to be on the same side as the non-dominant PcomA (19 patients, *P* < 0.001, McNemar’s test). Only 4 subjects had the dominant A1 and dominant PcomA on the same side.

### Comparison of vessel diameters and flow in different subgroups

#### AA group

**Fig. 3:**
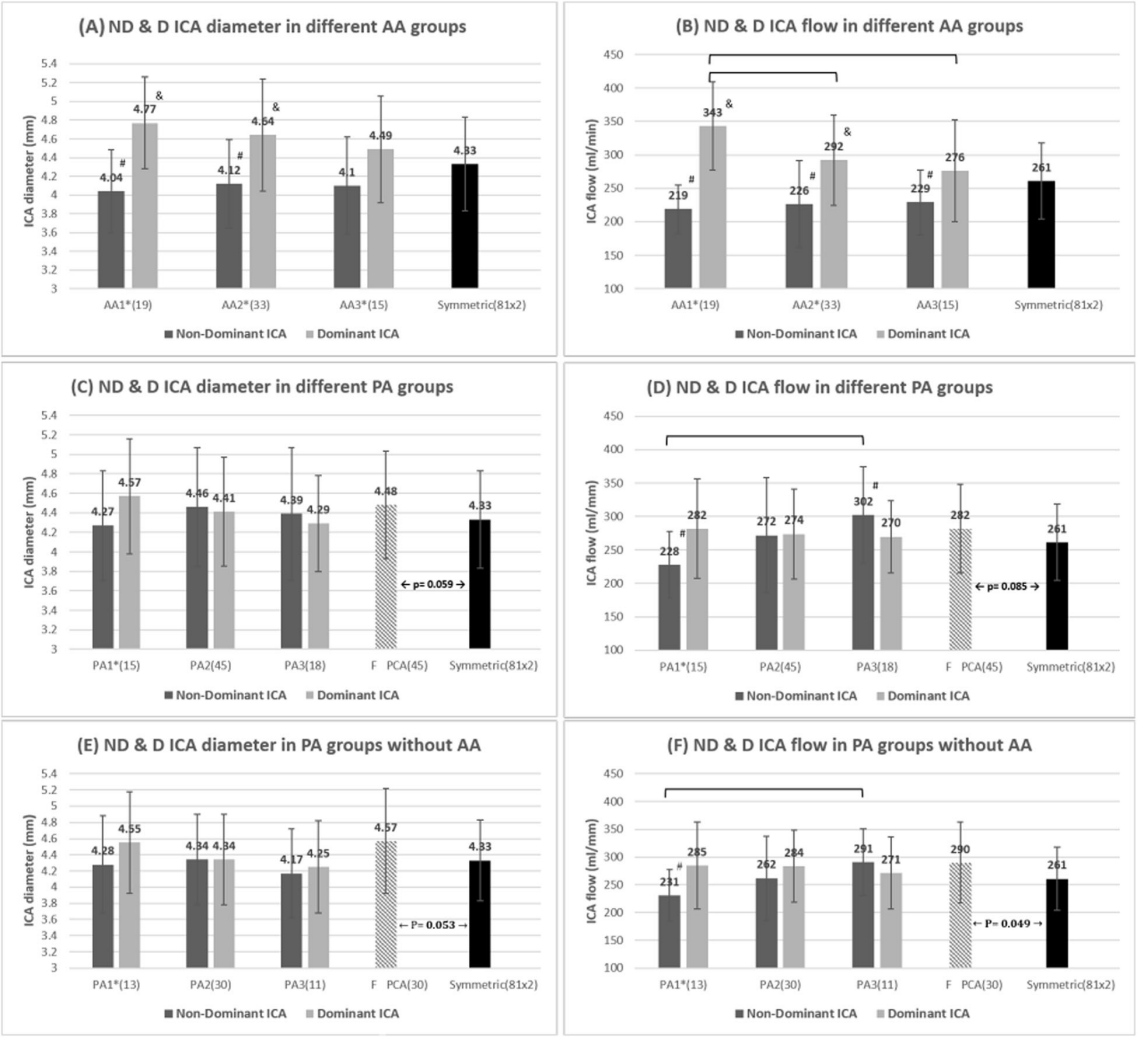
Vessel diameter and flow in each group of COW variants. (**a**) Diameter and (**b**) Flow volume of the non-dominant and dominant ICAs in the different AA groups; (**c**) Diameter and (**d**) Flow volume of the non-dominant and dominant ICAs in the different PA groups; (**e**) Diameter and (**f**) Flow volume of the non-dominant and dominant ICAs in different PA groups without A1 asymmetry.* Significant difference between non-dominant and dominant ICA in a certain subgroup (*P* < 0.05). # Significant difference between non-dominant ICA of a certain subgroup and ICA of the symmetric subgroup (*P* < 0.05). & Significant difference between dominant ICA of a certain subgroup and ICA of the symmetric subgroup (*P* < 0.05). The cover bar indicates a significant difference between different COW subgroups. (*P* < 0.05)

When ACA asymmetry was present, there were significant differences in ICA diameter between dominant and non-dominant sides in the AA1, AA2, and AA3 groups (Fig. 3a). Compared with the symmetric group, the AA1 and AA2 groups had a significantly larger ICA diameter on the dominant side and a significantly smaller ICA diameter on the non-dominant side. Similarly, significant differences in bilateral ICA flow were also found in the AA1 and AA2 groups (Fig. 3b). The average bilateral ICA flow volume difference percentages of the AA1, AA2, and AA3 groups were 42.9, 30.7, and 29%, respectively (Table 2). A greater asymmetry between the bilateral A1 segments tended to be associated with a larger difference between the bilateral ICA flow volume.

**Table 2:** ICA flow and diameter parameters for each group of COW variants

	**All**	**Symmetric**	**A1 Asymmetry (AA group)**					**PcomA Asymmetry (PA group)**				
			**AA1**	**AA2**	**AA3**	**Non-AA**	***P*value**	**PA1**	**PA2**	**PA3**	**Non-PA**	***P*value**
Number of subjects	210	81 (38.6%)	19 (9%)	33 (15.7%)	15 (7.1%)	143 (68.1%)		15 (7.1%)	45 (21.4%)	18 (8.6%)	132 (62.9%)	
ICA-PDF	6.9 ± 16.6	−0.1 ± 7.9	30.8 ± 22.4	17.4 ± 20.3	9.5 ± 16.4	1.0 ± 9.1	< 0.0001	7.3 ± 14.8	6.2 ± 13.3	7.3 ± 22.5	7.0 ± 17.0	0.993
ICA diameter difference (mm)	0.37 ± 0.32	0.22 ± 0.22	0.76 ± 0.33	0.55 ± 0.38	0.48 ± 0.38	0.26 ± 0.22	< 0.0001	0.40 ± 0.29	0.41 ± 0.34	0.40 ± 0.37	0.34 ± 0.31	0.655
ICA flow difference (ml/min)	65.9 ± 51.6	51.0 ± 38.8	124.6 ± 73.2	76.9 ± 45.8	76.2 ± 62.2	54.6 ± 42.0	< 0.0001	65.1 ± 47.3	64.0 ± 54.2	72.9 ± 53.4	65.6 ± 51.5	0.943
ICA flow difference percentage (%)	24.5 ± 17.8	19.6 ± 14.3	42.9 ± 24.1	30.7 ± 18.6	29.0 ± 19.0	20.2 ± 14.2	< 0.0001	25.0 ± 14.9	22.5 ± 16.0	25.5 ± 18.0	25.1 ± 18.7	0.852

#### PA group & F-PCA

A significant difference in bilateral ICA diameter and flow was only found in the PA1 group with unilateral F-PCA and no contralateral PcomA. If an F-PCA was present, there was a trend for the ipsilateral ICA to have a larger diameter and higher flow volume as compared with the symmetric group (Fig. 3c, d). However, there was no significant difference between these 2 groups concerning diameter (*P* = 0.059) and flow volume (*P* = 0.085). On the other hand, the difference between ICA flow ipsilateral to the F-PCA and in the symmetric group reached statistical significance (*P* = 0.049) after the exclusion of 15 patients with an F-PCA with concomitant A1 asymmetry (Fig. 3e, f).

### Association of ICA diameter with ICA flow

**Fig. 4:**
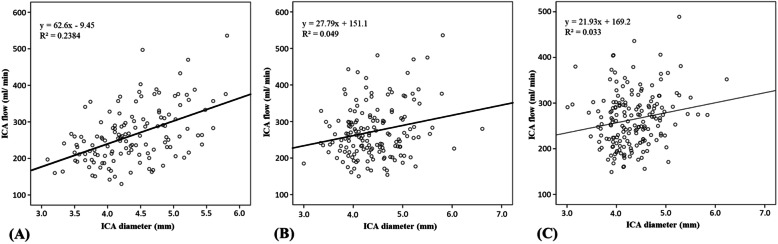
Linear regression analysis of ICA diameter and ICA flow in each group of COW variants. (**a**) AA group, (**b**) PA group, and (**c**) symmetric group

**Fig. 5:**
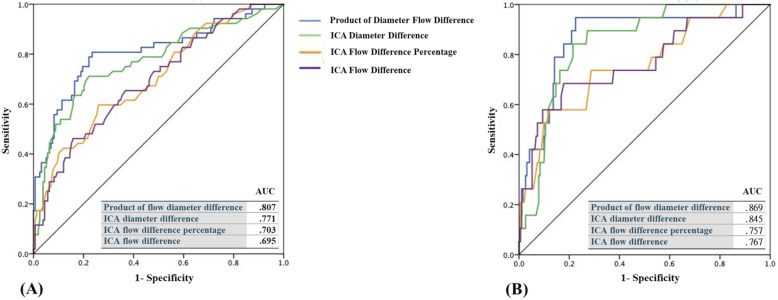
ROC curve of all parameters of ICA diameter and flow difference of different AA groups. (**a**) AA1 & AA2 vs others and (**b**) AA1 vs others

A moderately positive linear correlation between ICA diameter and ICA flow in the AA group (R^2^ = 0.238) was noted (Fig. 4a) According to the linear equation, there was a 62.6 ml increase in the ICA flow volume per 1 mm increase in the ICA diameter. Based on the average ICA flow volume of 265.9 ml/min in the AA group, it implied a change of 23.5% of the ICA flow volume per 1 mm change in the ICA diameter. On the other hand, no significant association was noted between ICA diameter and flow volume in either the PA (Fig. 4b) and symmetric groups (Fig. 4c). Carotid diameter and flow differences for each group of COW variants are shown in Table 2. All parameters, including ICA diameter difference, ICA flow difference, ICA flow difference percentage, and product of bilateral ICA diameter and flow difference (ICA-PDF) of each AA group exhibited significant differences. Among them, ICA-PDF had the highest area under the ROC curve (AUC = 0.807) for discriminating the AA1 and AA2 groups from the others, followed by ICA diameter difference (AUC = 0.771), ICA flow difference percentage (AUC = 0.703), and ICA flow difference (AUC = 0.695) (Fig. 5a). For the prediction of A1 absence, ICA-PDF and ICA-diameter difference exhibited even higher accuracy for discriminating the AA1 group from the other groups (Fig. 5b). The optimal cutoff value for bilateral A1 asymmetry ≥50% was a PDF = 4.31 with a sensitivity of 0.81 and specificity of 0.76.

## Discussion

This study attempted to clarify the effect of asymmetric COW variants on carotid flow changes and proposed an easy estimate of the representative carotid flow volume. In the present study, 52 of the 210 cases (34.7%) had bilateral A1 asymmetry ≥50%, in whom there was significantly greater dominant ICA flow with larger dominant ICA diameter, and lower non-dominant ICA flow with smaller non-dominant ICA diameter, as compared with the symmetric group. There was a moderately positive association between ICA vessel diameter and ICA flow volume in the AA groups, with a 1 mm increment in vessel diameter corresponding to a 62.6 ml increment of flow volume. Moreover, an ICA-PDF cutoff value of 4.31 was found to be a useful predictor of bilateral A1 asymmetry ≥50%.

While the COW serves as an important intracranial collateral pathway, there are multiple incomplete or asymmetric variants with variable prevalence according to different classification criteria, including A1 absence (2.6–15.4%; 9% in our study), A1 hypoplasia (2.6–28.9%; 15.7% in our study), true F-PCA without a P1 segment (0.9–29.5%; 10.7% in our study), and F-PCA with P1 hypoplasia (4.5–37.2%; 4.5% in our study) [9–12, 14, 15, 17, 18, 21–23]. In our study, we used a bilateral A1 and PcomA diameter ratio difference of 50% to define asymmetry rather than the usual criteria using an absolute vessel diameter of 1.0 mm as a cutoff value. Based on reports indicating the mean diameters of the A1 segment and the P2 segment are 2 mm [9, 11], a bilateral vessel diameter ratio difference of 50% equals a 1 mm cutoff value for the hypoplastic vessels. This vessel diameter ratio had been used in several studies focused on the effect of inflow inequality on cerebral aneurysms [23–26].

Our study showed a mean individual carotid flow difference percentage of 42.9% in the AA1 group. The result was consistent with several previous studies showing that the carotid flow ipsilateral to A1 aplasia is significantly lower than that of the contralateral side, with the flow difference percentage ranging from 34 to 50% [15–18]. Moreover, we also demonstrated that there was a significantly higher individual carotid flow difference percentage (30%) when A1 asymmetry was present. Since each A1 segment carries about 10% of the total brain flow to the ipsilateral hemisphere [17, 24], A1 segment asymmetry indicates redistribution of bilateral A1 flow and is crucial for computational hemodynamic studies for 2 reasons. First, A1 hypoplasia is associated with AcomA aneurysms [1, 13, 23, 27]. This relation can be explained by hemodynamic studies using either an experimental design with an A1 diameter ratio of ≥50% [25], or patient-specific 3D geometry with an unequal A1 inflow [28]. Both types of studies [25, 28] showed elevated intra-aneurysmal wall shear stress in the setting of A1 hypoplasia or unequal flow that would trigger AcomA aneurysm formation. Our results further emphasized that A1 asymmetry, even with a diameter difference of < 50%, could also cause unequal inflow and might be associated with Acom aneurysm formation. Second, several studies have indicated the importance of using patient-specific inflow boundary conditions to obtain reliable computational fluid dynamic results, especially for aneurysms with more than 2 inflow avenues, such as AcomA aneurysms [3, 19, 25, 28, 29]. Venugopal et al. [19] showed that the wall shear stress distribution on an aneurysm surface is sensitive to the bilateral A1 flow ratio and flow rate by using different inflow boundary conditions for a patient-specific AcomA aneurysm geometry with an original flow ratio of 1.87. Similarly, Karmonik et al. [29] reported that changes in the flow distribution of bilateral A1 segments could cause variations of the average wall shear stress as high as 43%, again using a patient-specific AcomA aneurysm model with an original flow ratio of 1.72. On the other hand, blood flow changes of the parent artery would not change the characterization of the intra-aneurysmal flow pattern substantially in the setting of a side-wall aneurysm/terminal aneurysm or AcomA aneurysm with relatively symmetric A1 segments [30]. In our study, we proposed a linear equation between the ipsilateral ICA diameter and ICA flow volume to provide a representative inflow boundary condition for the numerical simulation while A1 asymmetry is present.

As for PcomA asymmetry, only the PA1 group exhibited a significant carotid flow and diameter difference, but to a lesser extent of 25%. A significantly higher carotid flow with an F-PCA compared with the symmetric group was only found after the exclusion of coincident A1 asymmetry. When A1 asymmetry occurred simultaneously with PcomA asymmetry (23 cases), a non-dominant A1 (19 cases) was more frequently on the same side of the dominant PcomA. A PcomA usually serves as a conduit connecting the anterior and posterior circulation to provide a collateral pathway when there is proximal vessel comprise or there is an incomplete COW. The average net flow of a PcomA is usually low, and in an anterior to posterior direction, and accounts for about 5% of the ipsilateral carotid flow [31]. Considering all the aforementioned findings, it is reasonable that PcomA asymmetry has little influence on carotid flow changes.

In all AA groups and the PA1 group, there was a consistent relation between dominant and non-dominant carotid diameter and flow, i.e., there was larger vessel diameter and higher carotid flow on the dominant side. This finding is consistent with a “form-function” relation, wherein the form (anatomy: vessel radius) proportionately informs its function (physiology: blood flow). This concept has been extensively applied in the study of coronary artery disease [32, 33]. Similar findings have also been reported in studies of carotid flow, such as a small carotid diameter ipsilateral to A1 absence [21], and a linear relationship between whole brain volume and cerebral blood flow [16]. Cebral et al. [34] studied the flow-area relation in the carotid arteries of 11 healthy adults using the least-squares method for curve fitting and reported an average relative error between the predicted and the measured ratio to be 20%. This relation is also reflected in our finding of a moderately positive linear correlation (Fig. 4a) between carotid diameter and carotid flow in the AA groups (r^2^ = 0.238). Despite a significant difference in carotid diameter and flow between each of the AA groups, there was also a large standard deviation in each parameter owing to large individual variances. To cancel out the individual variances (Table S1 in the supplement) and amplify the flow-diameter difference in the subjects with A1 asymmetry (Figure S1 in the supplement), we proposed the product of bilateral ICA diameter and flow difference (ICA-PDF) as a potential discriminator. Recognition of an incomplete COW could offer stroke risk stratification in patients vulnerable to proximal artery compromise, such as those receiving cardiovascular surgery or carotid artery trapping [35].

Several limitations of our study should be addressed. First, the majority of subjects were middle-aged (40–60 years old, 64%) healthy Han-Chinese adults. The progressive decline of cerebral blood flow at a rate of 3 ml per year has been also reported [36]. The estimate of carotid flow in younger or aged populations might need adjustment. Second, it’s hard to assure the bright vessel lumen on TOF-MRA reflecting the “true” vessel size, especially when scanning a hypoplastic vessel. Moreover, the vessel diameter was calculated by only 1 reader. Thus, to minimize the measurement error of small-sized A1 segments and PcomA (< 3 mm), the vessel diameter ratio compared to the contralateral A1 or ipsilateral P2 segment, rather than the exact vessel diameter, was used for COW classification. It was reflected by the almost perfect reproducibility of categorical COW calcification in the 13 excluded subjects (Cohen K coefficient values of 0.85 ~ 0.96). Third, there was no validation performed for the estimate of carotid flow by the ipsilateral carotid diameter in A1 asymmetry and patients with specific neurovascular diseases such as cerebral aneurysms. Validation with another dataset, or with prospective study might be helpful.

## Conclusion

A1 asymmetry plays an important role in ICA flow distribution, contributing to bilateral unequal carotid inflow and significant carotid diameter difference. When A1 asymmetry is present, there is a moderately positive linear correlation between carotid diameter and carotid flow, with a 1 mm increment in vessel diameter corresponding to a 62.6 ml increment of flow volume. Our study improved the understanding of the association of bilateral ICA flow in healthy adults with asymmetric COW variants. Our results can potentially be applied to improve the accuracy of numerical simulation by the adjustment of the inflow boundary conditions according to the COW variants.

## Supplementary information

**Additional file 1: Figure S1.**ICA diameter difference and ICA flow difference percentage for each AA subgroup and non-AA subgroup.

**Additional file 2: Table S1.** ICA flow & diameter parameters for each AA subgroup and non-AA subgroup.

## Data Availability

The datasets used and analysed during the current study are available from the corresponding author on reasonable request.

## Abbreviations

COW: Circle of Willis
ICA: Internal carotid artery
ACA: Anterior cerebral artery
AcomA: Anterior communicating artery
A1: The 1st segment of anterior cerebral artery
AA: A1 asymmetry
PcomA: Posterior communicating artery
P1: The 1st segment of posterior cerebral artery
PA: P1 asymmetry
F-PCA: Fetal posterior cerebral artery
TOF: Time-of-flight
MRA: Magnetic resonance angiography
TAV: Time-average flow velocity
DRD: Diameter ratio difference
ICA-PDF: Product of bilateral ICA diameter and flow volume difference
