# Supplementary figures and images for: Correlation of internal carotid artery diameter and carotid flow with asymmetry of the circle of Willis
Source: BMC Neurol. 2020 Jun 20;20:251. doi: 10.1186/s12883-020-01831-z (PMC7305584; doi:10.1186/s12883-020-01831-z)

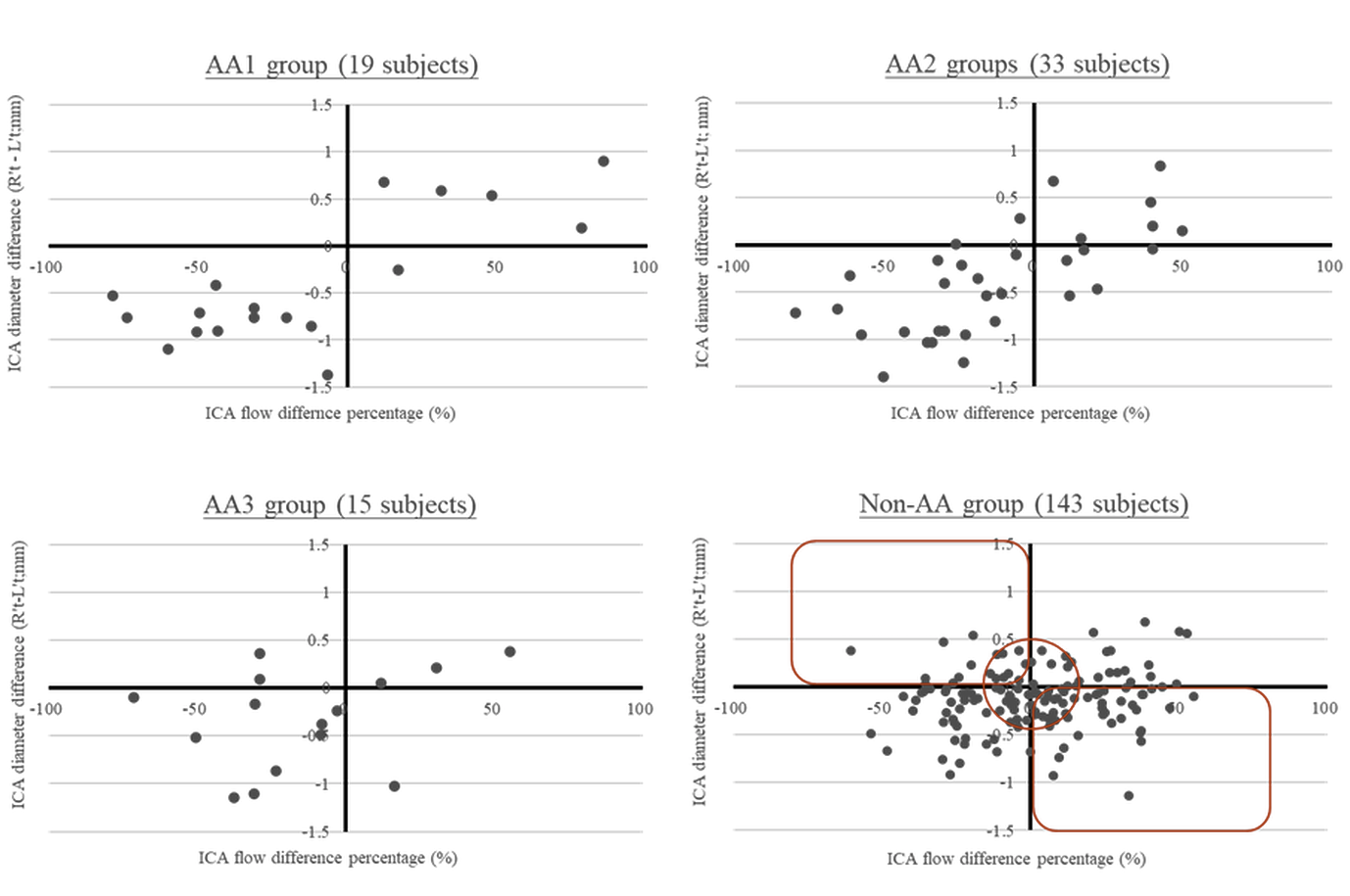

Supplement: Supplementary file 1 — Additional file 1: Figure S1.ICA diameter difference and ICA flow difference percentage for each AA subgroup and non-AA subgroup. [file 12883_2020_1831_MOESM1_ESM.tiff]
