# Supplementary material for: Correlation of internal carotid artery diameter and carotid flow with asymmetry of the circle of Willis
Source: BMC Neurol. 2020 Jun 20;20:251. doi: 10.1186/s12883-020-01831-z (PMC7305584; doi:10.1186/s12883-020-01831-z)
Supplement: Supplementary file 2 — Additional file 2: Table S1. ICA flow & diameter parameters for each AA subgroup and non-AA subgroup. [file 12883_2020_1831_MOESM2_ESM.docx]

Table S1. ICA flow & diameter parameters for each AA subgroup and non-AA subgroup.

|  | AA1 | AA2 | AA3 | Non-AA | P value |
| --- | --- | --- | --- | --- | --- |
| Number of subjects | 19 (9%) | 33 (15.7%) | 15 (7.1%) | 143 (68.1%) |  |
| ICA-PDF | 30.8 ± 22.4 | 17.4 ± 20.3 | 9.5 ± 16.4 | 1.0 ± 9.1 | <0.0001 |
| ICA diameter difference (mm) | 0.76 ± 0.33 | 0.55 ± 0.38 | 0.48 ± 0.38 | 0.26 ± 0.22 | <0.0001 |
| ICA flow difference (ml/min) | 124.6 ± 73.2 | 76.9 ± 45.8 | 76.2 ± 62.2 | 54.6 ± 42.0 | <0.0001 |
| ICA flow difference percentage (%) | 42.9 ± 24.1 | 30.7 ± 18.6 | 29.0 ± 19.0 | 20.2 ± 14.2 | <0.0001 |
| Dominant ICA flow (ml/min) | 343 ± 66.3 | 292 ± 67.7 | 276 ± 75.7 | 294 ± 63.4 | 0.010 |
| Dominant ICA diameter (mm) | 4.77 ± 0.49 | 4.64 ± 0.60 | 4.49 ± 0.57 | 4.48 ± 0.54 | 0.094 |
| Non-dominant ICA flow (ml/min) | 219 ± 35.8 | 226 ± 64.9 | 229 ± 48.5 | 240 ± 49.6 | 0.239 |
| Non-dominant ICA diameter (mm) | 4.04 ± 0.44 | 4.12 ± 0.47 | 4.10 ± 0.52 | 4.21 ± 0.48 | 0.342 |
